# Being a grump only makes things worse: a transactional account of acute stress on mind wandering

**DOI:** 10.3389/fpsyg.2013.00730

**Published:** 2013-11-12

**Authors:** Melaina T. Vinski, Scott Watter

**Affiliations:** Department of Psychology, Neuroscience and Behaviour, McMaster UniversityHamilton, ON, Canada

**Keywords:** transactional model of stress, mind wandering, negative mood, coping strategies, pupil diameter

## Abstract

The current work investigates the influence of acute stress on mind wandering. Participants completed the Positive and Negative Affect Schedule as a measure of baseline negative mood, and were randomly assigned to either the high-stress or low-stress version of the Trier Social Stress Test. Participants then completed the Sustained Attention to Response Task as a measure of mind-wandering behavior. In Experiment 1, participants reporting a high degree of negative mood that were exposed to the high-stress condition were more likely to engage in a variable response time, make more errors, and were more likely to report thinking about the stressor relative to participants that report a low level of negative mood. These effects diminished throughout task performance, suggesting that acute stress induces a temporary mind-wandering state in participants with a negative mood. The temporary affect-dependent deficits observed in Experiment 1 were replicated in Experiment 2, with the high negative mood participants demonstrating limited resource availability (indicated by pupil diameter) immediately following stress induction. These experiments provide novel evidence to suggest that acute psychosocial stress briefly suppresses the availability of cognitive resources and promotes an internally oriented focus of attention in participants with a negative mood.

## INTRODUCTION

The experience of stress is pervasive. In 2011, 23.6% of adult Canadians reported experiencing extreme stress in daily life (Statistics Canada, 2011). With chronic stress linked to immunological (e.g., Herbert and Cohen, 1993; Kiecolt-Glaser et al., 1996; Cohen et al., 2001; Esch et al., 2002a; Segerstrom and Miller, 2004, for reviews), cardiovascular (e.g., Harlan, 1981; Esch et al., 2002b; Bunker et al., 2003), and neurodegenerative disease (e.g., Gilad et al., 1990; Gilad and Gilad, 1995), as well as poor mental health (e.g., Negrão et al., 2000; Vaidya, 2000; Raison and Miller, 2003), an abundance of research has emerged with the aim of mapping both the psychological and physiological determinants of the body’s response to stress.

Transactional models of stress (Lazarus and Folkman, 1984) are multidimensional representations that emphasize the reciprocal relation between state and stressor on behavior. Transactional models provide an algorithm that characterizes stress response based on an individual’s appraisal of environmental demands and subsequent choice of coping strategy (Lazarus, 1966, 1999; Stokes and Kite, 1994, 2000; Zeidner and Endler, 1996; Hammond, 2000). Appraisal mechanisms evaluate the degree of personal relevance within the stressful context, while coping mechanisms regulate whether the individual engages in either a task-focused or emotion-focused strategy (Lazarus and Folkman, 1984). A task-focused coping strategy operates to prevent distraction and maintain successful task performance. An emotion-focused coping strategy is intimately tied to self-regulation and self-referential mentation, and facilitates the prioritization of processing internal worries at the expense of concurrent task performance (e.g., Matthews and Desmond, 2002).

While transactional models provide a framework for developing a state-mediated model of stress, transactional theories lack specific predictions on information processing (Matthews and Campbell, 2009). Matthews (2001) proposed two separate transactional frameworks for modeling the effects of stress on cognition in order to address this gap in the literature. Cognitive-adaptive transactions aim to model the stress response as a function of the allocation of attention and energy, where behavior is driven by goals and intentions and operates to direct attention as a function of coping strategy. Those who enlist a task-focused coping strategy are therefore more likely to allocate attention toward task supervision in efforts to maintain successful task performance, while those who enlist and an emotion-focused coping strategy are more likely to allocate attention toward self-referential mentation (and therefore are more likely to perform poorly on the task). This framework aligns with previous reports that stress promotes the select processing of information relevant to an individuals’ current goal state (Chajut and Algom, 2003; Kofman et al., 2006). Bio-cognitive transactions on the other hand, are modulated by fluctuations in both neural and cognitive architectures that directly influence the availability of attentional resources, where the term resources refers to the capacity of the executive attention network to process information (see Kane and Engle, 2002). Indeed, stress has been shown to deplete working memory capacity independent of task difficulty (Schoofs et al., 2008) and modulate cognitive control functioning (Alexander et al., 2007; Steinhauser et al., 2007; Liston et al., 2009). The two conceptual frameworks are therefore inherently linked and complementary: bio-cognitive transactions model the limitation of attentional resources during periods of stress, while cognitive-adaptive transactions model the allocation of attention within the constraints of resource availability. The current work aims to investigate the effect of stress on mind-wandering behavior, using both a cognitive-adaptive (Experiment 1) and bio-cognitive (Experiment 2) parameters to provide a comprehensive account of the effects.

In the situation of an emotion-focused coping strategy, attention is allocated toward the internal world rather than the task environment. Often referred to as mind-wandering episodes (Smallwood et al., 2003a, 2003c; for a review, see Smallwood and Schooler, 2006**)**, these mental diversions are associated with poor task performance (see Smallwood and Schooler, 2006) and typically occur during well-practiced or mundane tasks (e.g., Teasdale et al., 1995). While mind-wandering behavior consumes almost half of our conscious experience in daily life (Killingsworth and Gilbert, 2010), such behavior occurs more frequently in some populations relative to others. For example, clinically depressed (e.g., Carriere et al., 2008) and dysphoric populations (Smallwood et al., 2007) are more likely to engage in mind-wandering behavior compared to healthy controls. A similar bias toward internal processing has been observed following negative mood induction in healthy individuals. Smallwood et al. (2009) found that participants induced with a negative mood were more likely to engage in task-irrelevant thought and were less able to effectively disengage from such thoughts relative to controls. Smallwood and O’Connor (2011) observed a similar increase in mind-wandering behavior following negative mood induction, and report a retrospective bias during episodes of off-task thought in the negative mood group relative to controls.

Negative mood has also been implicated as a critical determinant of coping strategy in studies of stress (Parker and Brown, 1982). Coyne et al. (1981) compared coping mechanisms in stressful situations between depressed and non-depressed populations over a 1-year period. Using self-report as the primary measure, the authors report that being in a depressive state promotes an emotion-focused coping following a stressful experience. Depressive states have also been associated with a heightened perception of what is at stake in a stressful situation (e.g., self-esteem and well-being) relative to controls (Lazarus and Folkman, 1984), and is associated with fewer available personal and social resources to mediate the effects of stress (Mitchell et al., 1983).

Negative mood appears to systematically bias the allocation of attention toward a self-referential mentation within both the mind wandering and stress literature. The current work therefore attempts to provide novel evidence for a cognitive-adaptive (Experiment 1) and bio-cognitive (Experiment 2) account of stress on mind-wandering behavior, with negative mood isolated as the state variable within the transactional framework. We grouped participants based on their level of state negative mood, and exposed them to either a high-stress procedure or a low stress control procedure. In Experiment 1, we predicted that in the high negative mood group, the high-stress condition would induce an emotion-focused coping strategy and an allocation of attention toward the internal environment, resulting in an increase in mind-wandering behavior relative to the low stress condition. In Experiment 2, we aimed to replicate the behavioral effects of Experiment 1, and predict a limitation of resource availability (indicated by larger pupil diameter; Beatty, 1982) following participation in the high-stress condition for the high negative mood group relative to the low stress condition. Finally, the effect of stress on cognition has been shown to diminish over time (Elzinga and Roelofs, 2005; Oei et al., 2006), and as such we predict that stress effects are likely to be maximal immediately after stress induction (block 1) in both experiments. As a most direct test of these hypotheses, we used planned comparisons based on *a priori* predictions of high-stress experiences affecting high negative affect more adversely than low negative affect participants, immediately following stress induction, in addition to omnibus tests.

## EXPERIMENT 1

### MATERIALS AND METHODS

#### Participants

Participants (*n* = 124; 51 in the experimental group, 26 females; and 73 in the control group, 43 females) were undergraduate students from McMaster University. Participants were recruited using the University’s online experiment scheduling system and received partial course credit in exchange for their participation.

#### Procedure

Participants spent approximately 1 h in the laboratory. After providing written informed consent, participants were asked to complete the Extended Positive and Negative Affect Schedule (PANAS-X; Watson et al., 1988). The PANAS-X provides a subscale measure of an individual’s level of state positive and negative mood, and was used as a baseline measure of state mood prior to the stress induction procedure. In the current work, our predictions were inclusive to participants’ scores on the negative subscale only. As a result, participants’ scores on the positive subscale were not included in the analyses.

Participants then randomly assigned to either the “high-stress” experimental condition (Kirschbaum et al., 1993) or the “low stress” control condition (Het et al., 2009) of the Trier Social Stress Test (TSST), a standardized paradigm that reliably activates the body’s stress response (Dickerson and Kemeny, 2004; Kuhlmann and Wolf, 2005). Both conditions had two phases. The first phase included an anticipation period, where participants were asked to sit in a waiting room alone for 5 min. This first phase was the same for both conditions. In the second phase, participants were moved into a second experiment room. In the high-stress condition, participants were required to complete complex verbal arithmetic and to give an impromptu speech (on their career plan following completion of their undergraduate degree) in front of a three-member confederate panel. Participants were informed that their performance was being taped for further consideration. In the low stress condition, participants were required to count aloud in intervals of 15 without a panel for the verbal arithmetic task, and informally talk about their favorite movie or experience in front of a single panel member for the speech task (as per Kirschbaum et al., 1993**).**

Participants were then guided to another experiment room where they completed the Sustained Attention to Response Task (SART; Robertson et al., 1997) as a measure of mind-wandering propensity. The SART is a go/no-go task that requires participants to respond quickly and accurately on non-target trials, and withhold their response on infrequent target trials. In the current work, target probability was 20%, with the non-target stimuli being the numbers 0 through 9 and the target stimulus being the number 3 (for a review, see Smallwood and Schooler, 2006). Probe trials immediately followed target trials to assess the participants’ conscious experience of mind-wandering behavior. The probe read “Stop! Where was your attention focused immediately prior to this question?” with an alternative forced choice response format of either “my attention was focused on the task” or “my attention was not focused on the task.” The proportion of off-task thought was calculated by dividing the number of off-task thought probes by the total number of thought probes (Vinski and Watter, 2012). The experiment was presented to participants using E-prime software (Schneider et al., 2002). Participants were shown two blocks of 179 stimuli, with stimuli presented for 700 ms and an inter-stimulus interval of 2000 ms. The stimuli were presented in black print on a white background.

Response time variability on non-target trials, known as the response time coefficient of variability (RTCV, calculated as reaction time SD divided by the mean), and the proportion of inappropriate responding on target trials (error rate) were calculated as the behavioral indicators of task inattention (see Smallwood and Schooler, 2006). Response time variability has been validated as a measure of natural variation in attentional focus throughout task performance and has emerged as an increasingly relevant behavioral measure within the mind wandering literature (Cheyne et al., 2009). Error rate is indicative of failures in top-down supervision of task performance (Robertson et al., 1997; Manly et al., 1999), and has traditionally been utilized as a measure of off-task focus (Smallwood and Schooler, 2006; Smallwood et al., 2007). However, convergent evidence suggests that not all performance errors provide direct evidence of inattention (e.g., Peebles and Bothell, 2004; Helton, 2009), with error rates mediated by both signal salience and the speed-accuracy trade off (See et al., 1995; Manly et al., 1999) and vulnerable to impulsivity (e.g., Helton, 2009). Correlations were therefore computed between dependent measures for the first block of the experiment and across all experimental blocks to validate that the three dependent measures reflect the same construct. Correlations are shown in **Table 2**. The proportion of times participants failed to respond on non-target trials, typically referred to as the omission rate (Cheyne et al., 2009), was not included as a dependent measure due to the limited time in which participants were required to make their response on non-target trials (700 ms).

Retrospective probes were inserted following the first and second experimental block to provide a measure of thought content during mind-wandering episodes. Retrospective report of off-task thought has been validated with both intermittent thought probes and response latencies during task performance (Smallwood et al., 2004), and therefore provides an opportunity to collect an additional measure of attentional focus during task performance. Participants were given 3 min to type the content of their thoughts during the experimental block immediately prior. Participants were reassured that the report would be confidential and were encouraged to be as detailed in their report as possible. Thought content was coded based on three parameters: tense, valence, and focus. Tense refers to whether participants’ thoughts were focused on episodes that occurred prior to the experimental session (past), episodes that occurred within the experimental session (present), or expectation of future episodes (future; modified from criterion defined by Smallwood et al., 2011b). Valence refers to the affective tone of participants’ thoughts. Content was coded as either positive, negative, neutral, or a select combination of either affective valence. Focus refers to the focus of participants’ thoughts with respect to the experimental paradigm. Content was coded as either related to the SART (task-related), to their experience during the TSST (TSST-related) or unrelated to the experimental session (task-unrelated; see Smallwood et al., 2003b for a similar coding criterion). If participants reported thoughts that coincided with more than one level of a given factor (for example, reported both positive and negative thoughts within the same retrospective probe) then the participant was classified as “Other.”

Two assistants in the laboratory were recruited to code the thought probes, and were extensively debriefed on the coding scheme prior to completing the classification. A cross-coding validation procedure was employed to validate the categorization of the participants’ retrospective reports, whereby the two coders compared classification for each subject (as per Smallwood et al., 2003a). Discrepancies between coders were resolved through discussion, and a third party was included if the case of indecision. In the current work, all discrepancies were resolved through discussion and no third party mediation was necessary. Coders were blind to the experimental hypotheses and participant information. Participants were extensively debriefed following participation.

### RESULTS

**FIGURE 1:**
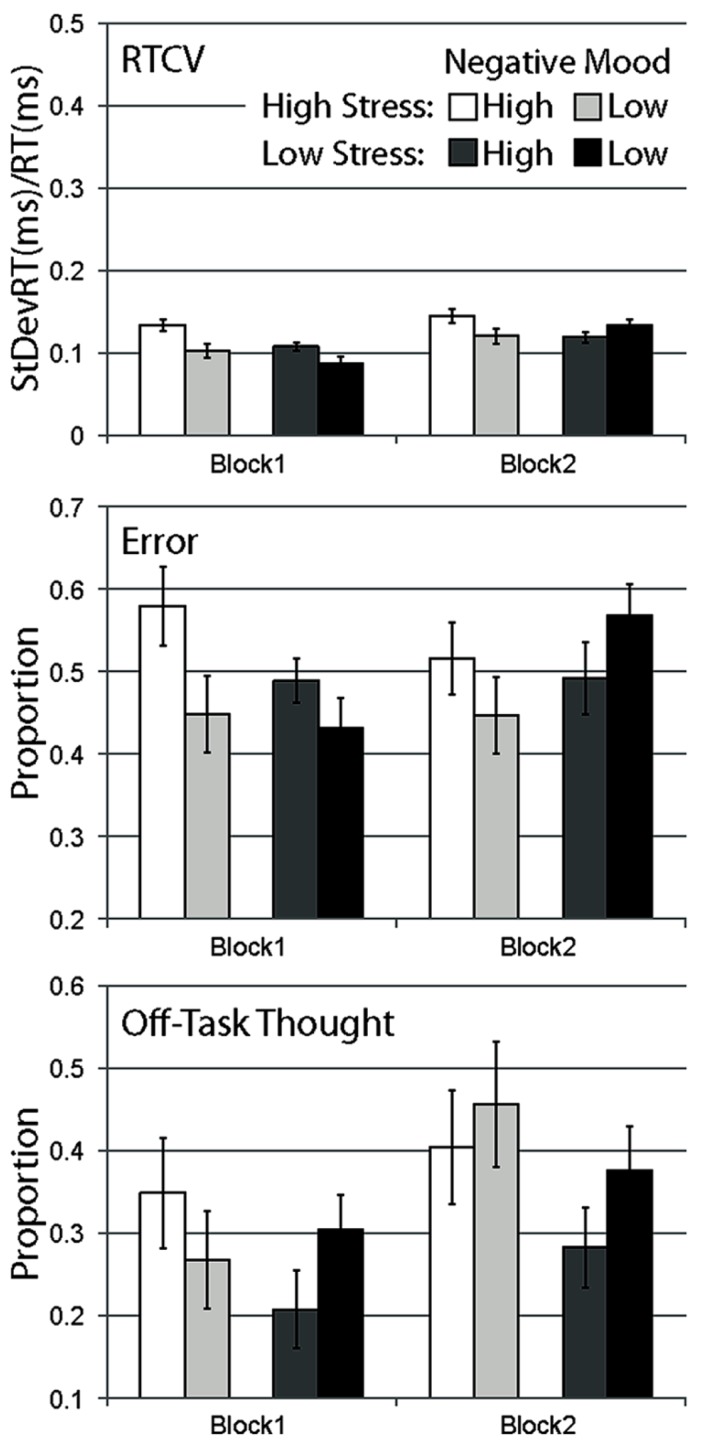
**Mean data for Experiment 1, separated by stress manipulation, negative mood group, and block.** Data are shown for RTCV (reaction time coefficient of variability, calculated as reaction time standard deviation divided by the mean), error (proportion of commission errors on no-go trials), and proportion of probe trials reporting off-task thought. Error bars represent standard errors.

The first set of analyses were designed to investigate the effects of stress on mind-wandering behavior across task duration, as a function of negative mood reported prior to stress induction. Behavioral (response time variability and error rate) and experience sampling data (self-report probes) were analyzed using mixed analysis of variance (ANOVA), with the within-subjects factor of *Block* (block 1, block 2) and the between subjects factors *Stress* (low, high) and *Baseline* *Negative Affect* (high, low). Participants were grouped into either the high mood group (*M =* 26.74, SD = 4.07) or low mood group (*M* = 15.91, SD *=* 3.45) using a median-split technique on the PANAS-X negative subscale scores ($\overset{∼}{x}$ = 21.7). All analyses were performed with negative affect included as a continuous variable. Because the results and significance of findings were not affected, we report median-split analyses only. In addition to our omnibus analyses, we conducted a small number of directional planned comparisons, focusing on high versus low negative mood participants in the high-stress condition in block 1 data. These planned comparisons were based on *a priori* predictions of greater stress-mediated disruption to focused performance in high negative affect participants that are likely to diminish over time. Mean data are shown in **Figure 1**.

#### Response time variability

A strong main effect of block was observed, with response time variability increasing across blocks for all conditions, *F*(1,120) = 24.648, *p* < 0.001. A main effect of stress, *F*(1,120) = 5.030, *p* < 0.05 and a main effect of negative mood was observed, *F*(1,120) = 6.307, *p* < 0.05. A significant interaction between negative mood and stress were observed, *F*(1,120) = 4.093, *p* < 0.05, with the high negative mood group more likely to engage in variable response time behavior relative to the low mood group during the high-stress condition, *F*(1,50) = 6.964, *p* < 0.05, but not for the low stress condition, *F*(1,50) = 0.204, *p* = ns. These findings support the observation that while both high-stress and high pre-existing negative mood produced more variable response behavior, the degree to which stress influenced response behavior was dependent on participants’ level of negative mood. A reliable interaction was also observed between block and negative mood, *F*(1,120) = 5.465, *p* < 0.05, with the high mood group exhibiting a more variable response behavior relative to the low mood group during the first experimental block, *t*(122) = 3.323, *p* < 0.01, but not the second, *t*(122) = 0.238, *p* = *ns*. The block by stress interaction did not reach significance, *F*(1,120) = 2.498, *p* = ns, nor did the three-way interaction, *F*(1,120) = 2.669, *p* = ns.

Considering our *a priori* expectation that stress effects should be maximal immediately following stress induction, we examined data from just our first experimental block. In the high-stress experimental group, high negative mood participants were significantly more likely to engage in more variable response behavior relative to low negative mood participants during the first experimental block, *t*(49) = 2.680, *p* < 0.01, one-tailed. High negative mood participants were also more likely to demonstrate a variable response time relative to the low negative mood group in the low stress condition, *t*(71) = 2.178, *p* < 0.05, one-tailed.

#### Error rate

In contrast to response time variability, there was no main effect of block on error rate data, *F*(1,120) = 0.776, *p* = ns, no main effect of stress, *F*(1,120) = 0.005, *p* = ns, and no main effect of negative affect, *F*(1,120) = 1.626, *p* = ns. Error rates were relatively larger for high-stress versus control participants in the first block, but more comparable in the second block, supported by the interaction of block with stress condition, *F*(1,120) = 5.786, *p* < 0.05. Similarly, error rates were relatively larger for high versus low negative affect groups in the first block, *F*(1,50) = 3.901, *p* = 0.054, but comparable in the second block, *F*(1,50) = 1.540, *p* = ns, supported by the interaction of block with negative affect, *F*(1,120) = 5.379, *p* < 0.05. The stress by negative affect interaction was not significant, *F*(1,120) = 2.410, *p* = ns, and there was no three-way interaction, *F*(1,120) = 0.725, *p* = ns.

Planned comparisons showed that in congruence with response time variability, in the high-stress experimental group, high negative mood participants were significantly more likely to make errors relative to low negative mood participants during the first experimental block, *t*(49) = 1.966, *p* < 0.05, one-tailed. This effect of negative mood failed to reach significance in the low stress group, *t*(71) = 1.265, *p* = ns, one-tailed.

#### Self-report of off-task thought

As the subjective counterpart to the behavioral measures of task inattention, self-report probes provide the opportunity for online experience sampling throughout task performance (Smallwood and Schooler, 2006). Effects on self-report data were less robust than for behavioral dependent measures. There was a strong main effect of block, with all conditions showing greater proportions of reported off-task thought over blocks, *F*(1,120) = 13.917, *p* < 0.001. The main effect of stress, *F*(1,120) = 2.289, *p* = ns, the main effect of negative mood, *F*(1,120) = 0.623, *p* = ns, and the interaction between these two factors failed to reach significance, *F*(1,120) = 1.156, *p* = ns. There were also no significant interactions of stress and block, *F*(1,120) = 0.869, *p* = ns, negative mood and block, *F*(1,120) = 1.533, *p* = ns, and no three-way interaction, *F*(1,120) = 1.715, *p* = ns.

Planned comparisons of block 1 data for proportion of off-task thought did not show a difference for high versus low negative mood participants in the high-stress condition, *t*(49) = 0.906, *p* = ns, one-tailed.

#### Retrospective report of thought content

The next series of analyses were designed to investigate the content of participants’ mind-wandering episodes between high and low negative mood participants assigned to the high-stress condition. Retrospective thought content was analyzed using a Chi-square Test of Independence on the factors *Negative Mood* (low, high) as well as *Tense* (past, present, or future), *Valence* (positive, negative, or neutral), and *Focus* (task-related, TSST-related, and task-unrelated) during the first and second experimental block. Descriptive statistics for the first experimental block are shown in **Table 1**.

**Table 1:** Percentage of retrospective reports for each factor during the first experimental block, for both the low and high negative mood groups, across Experiments 1 and 2.

Factor	Level	Experiment 1		Experiment 2	
		Low negative mood	High negative mood	Low negative mood	High negative mood
Tense	Past	0.05	0.11	0.08	0.30
	Present	0.60	0 50	0.58	0.50
	Future	0.00	0.00	0.33	0.20
	Other	0.35	0.39	0.01	0.00
Valence	Positive	0.00	0.00	0.00	0.00
	Neutral	0.95	0.78	0.50	0.40
	Negative	0.05	0.22	0.33	0.60
	Other	0.00	0.00	0.12	0.00
Focus	Task-related	0.40	0.05	0.00	0.00
	Stressor-oriented	0.00	0.28	0.33	0.70
	Task-unrelated	0.60	0.67	0.25	0.30
	Other	0.00	0.00	0.42	0.00

During the first block, negative mood was significantly related to Focus, χ^2^(2) = 6.190, *p* < 0.05, Cramer’s *V* = 0.404, with the high negative mood group more likely to entertain thoughts about the TSST and the low mood group more likely to entertain thoughts relating to task performance. The effect of negative mood on retrospective report failed to reach significance for Tense, χ^2^(2) = 1.035, *p* = ns, *V* = 0.165, and with participants only reporting either neutral or negative thoughts, Valence also failed to reach significance, χ^2^(1) = 2.459, *p* = ns, *V* = 0.254. Group differences failed to reach significance for Focus χ^2^(2) = 2.46, *p* = ns, or the remaining factors during the second experimental block (χ^2^ < 1).

### DISCUSSION

Findings suggest that for those who are in a negative mood, acute stress activates an emotion-focused coping strategy that fosters an inattentive state (variable response time and a boost in error rates) relative to those in the low negative mood group. Consistent with predictions, the observed effects were prominent during the first experimental block only.

The aim of Experiment 1 was to provide evidence for a cognitive-adaptive transactional account of stress on mind-wandering behavior. The present findings are compatible with such an account, with acute stress inducing a temporary stressor-oriented bias in attentional allocation relative to the low stress group, with performance deficits potentiated by the degree of negative mood reported prior to stress induction. To complement the cognitive-adaptive findings in Experiment 1, Experiment 2 was designed to provide evidence for a bio-cognitive account of stress on mind-wandering behavior.

## EXPERIMENT 2

Bio-cognitive transactions are mechanisms that are modulated by the fluctuation in both neural and cognitive architectures that influence availability of resources. Pupil dilation has been shown to reliably reflect resource utilization during cognitively demanding tasks regardless of the sensory domain under investigation (Beatty, 1982), with dilation systematically increasing as a function of cognitive load (Granholm et al., 1996; for a review, see Kahneman, 1973; Beatty, 1982). Experiment 2 was designed to replicate the behavioral and self-report trends observed during Experiment 1, while providing a psychophysiological measure of resource availability compatible with a bio-cognitive transactional theory of stress.

The first set of analyses were conducted to replicate the stress effects observed in Experiment 1, and investigate how the observed trends extended into a third experimental block. It was hypothesized that behavioral trends and retrospective reports would replicate the findings observed in Experiment 1, predicting that participants that report a high level of negative mood will be more likely to engage in mind-wandering behavior (high response time variability and error rates) in the high-stress condition relative to the low negative mood group, with maximal effects likely during the first experimental block. As per Experiment 1, we predict that task disengagement in the high negative mood group is due to the initiation of an emotion-focused coping strategy, and therefore expect that this group will also be more likely engage in ruminatory processing of the stressor when compared to the low negative mood group. In light of previous reports of reduced resource availability during episodes of stress in those with a negative disposition (Coyne et al., 1981; Lazarus and Folkman, 1984), we predict that individuals in the high negative mood group will also demonstrate a larger pupil diameter immediately following stress induction relative to the low negative mood group, a trend that would parallel the behavioral indicators of mind-wandering behavior observed during the first experiment. As per Experiment 1, correlations between dependent measures for the first experimental block and across all experimental blocks are shown in **Table 2**.

**Table 2:** Correlations Between SART-Dependent Measures During Block 1 And Across Task Performance, For Both Experiments 1 And 2.

	Dependent Measure	Experiment 1			Experiment 2		
		Rtcv	Error Rate	Self-Report Of Off-Task Thought	Rtcv	Error Rate	Self-Report Of Off-Task Thought
Performance During Block 1	Rtcv	–	0.367^*^	0.062	–	0.480^*^	0.136
	Error Rate	0.367^*^	–	0.101	0.480^*^	–	0.453^*^
	Self-Report Of Off-Task Thought	0.062	0.101	–	0.136	0.453^*^	–
Performance Across All Task Blocks	Rtcv	–	0.409^*^	0.133^*^	–	0.543^*^	0.109
	Error Rate	0.409^*^	–	0.048	0.543^*^	–	0.497^*^
	Self-Report Of Off-Task Thought	0.133^*^	0.048	–	0.109	0.497^*^	–

* p < 0.05.

### MATERIALS AND METHODS

#### Participants

Participants (*n* = 47; 23 in the experimental group, 13 females; and 24 in the control group, 15 females) were undergraduate students from McMaster University. Participants were recruited using the University’s online experiment scheduling system and received partial course credit in exchange for their participation.

#### Procedure

The experimental procedure was the same as Experiment 1. Participants completed the PANAS-X as a measure of baseline mood and were randomly assigned to either the control or experimental version of the TSST, and then completed the SART. While our predictions are exclusively focused on the first experimental block, we included a third block to provide a measure of group differences over a longer period of time. Participants were shown three blocks of 179 stimuli, with a 2000-ms inter-stimulus interval. Pupil diameter was measured using participants’ right eye using a head-mounted EyeLink II video eye tracking system (SR Research). Pupil diameter was sampled at 250 Hz continuously throughout SART performance, and the average pupil diameter within each trial was recorded, and then averaged across trials to provide a mean pupil diameter per block.

### RESULTS

**FIGURE 2:**
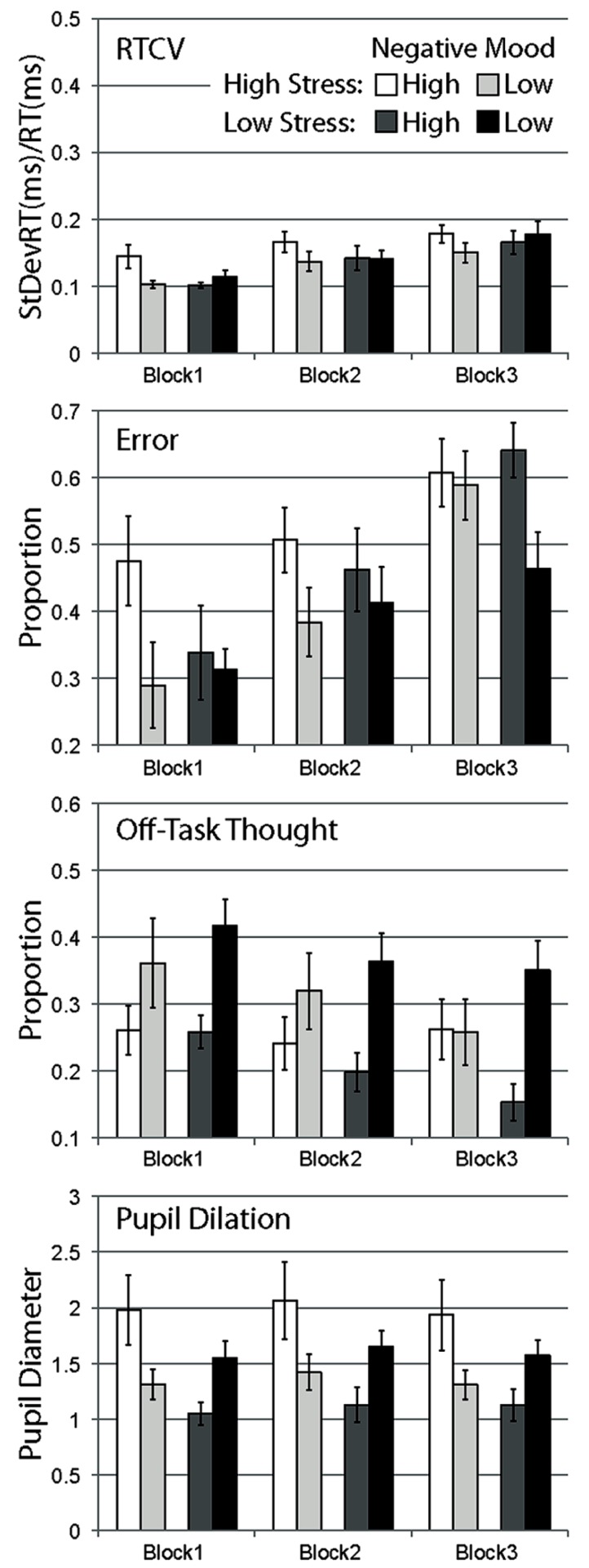
**Mean data for Experiment 2, separated by stress manipulation, negative mood group, and block.** Data are shown for RTCV (reaction time coefficient of variability, calculated as reaction time standard deviation divided by the mean), error (proportion of commission errors on no-go trials), proportion of probe trials reporting off-task thought, and pupil diameter. Error bars represent standard errors.

Behavioral, experience sampling, and pupillometric data were analyzed using a mixed ANOVA on the within-subjects factor *Block* (block 1, block 2, block 3) and the between subjects factors *Stress* (high, low) and *Negative Mood* (low, high). As per Experiment 1, participants were grouped into either the high mood group (*M =* 24.44, SD = 2.91) or low mood group (*M* = 16.37, SD = 2.79) using a median-split analysis ($\overset{∼}{x}$ = 19.4). As per Experiment 1, analyses with negative affect as a continuous variable yielded the same significance of results, and therefore median-split analyses are reported only. As in Experiment 1, in addition to our omnibus analyses, we conducted a small number of directional planned comparisons, focusing on high versus low negative mood participants in the high-stress condition in block 1 data. These planned comparisons were based on *a priori* predictions of greater stress-mediated disruption to focused performance in high negative affect participants that are likely to diminish over time. Mean data are shown in **Figure 2**.

#### Response time variability

Response time coefficient of variability was observed to increase over blocks for all conditions, supported by a strong main effect of block, *F*(2,86) = 28.055, *p* < 0.001. While RTCV scores were numerically largest for high negative affect participants in the high-stress group in all blocks, there was no main effect of stress, *F*(1,43) = 0.234, *p* = ns, no main effect of negative affect, *F*(1,43) = 0.903, *p* = ns, and no significant interaction between these two factors, *F*(1,43) = 2.364, *p* = ns. None of these effects interacted with block, all *F*s < 1.5.

Planned comparisons of block 1 data suggested that in the high-stress group, high negative mood participants showed more variable response behavior compared to low negative mood participants, *t*(20) = 2.369, *p* < 0.05, one-tailed, congruent with the pattern of data observed in Experiment 1. This effect of mood was not observed for participants in the low stress group, *t*(23) = 0.882, *p* = ns, one-tailed.

#### Error rate

Error rate was also observed to increase over blocks for all conditions, with a strong main effect of block, *F*(2,86) = 32.436, *p* < 0.001. In the first block, error scores appeared to be substantially larger for high negative affect participants in the high-stress condition compared to other groups. This difference appeared to diminish across subsequent blocks, with participants in each condition equally likely to make errors over time, with some apparent sparing of performance (relatively fewer errors) in the low negative affect, low stress group in the last block. This pattern of data was supported by a significant main effect of negative affect, *F*(1,43) = 4.215, *p* < 0.05, and the three-way interaction of stress, negative affect, and block, *F*(2,86) = 4.444, *p* < 0.05. No other effects were significant, *F*s < 0.7.

Planned comparisons of error data in block 1 demonstrate a higher error rate during task performance for high versus low negative mood participants in the high-stress condition, *t*(20) = 2.012, *p* < 0.05, one-tailed. This effect of mood was not observed for the low stress condition, *t*(23) = 0.384, *p* = ns, one-tailed.

#### Self-report of mind wandering

In contrast to the general pattern of performance over time, and to self-report of mind wandering in Experiment 1, proportion of self-report of mind-wandering episodes in Experiment 2 was observed to generally increase over blocks, *F*(2,86) = 7.945, *p* < 0.01, with a strong linear trend, *F*(1,43) = 9.056, *p* < 0.01. Compared to this general increase in mind wandering over blocks observed in most conditions, the high stress, high negative mood condition appeared relatively consistent over blocks. This pattern of data was partially supported by a marginal three-way interaction of stress, negative mood and block, *F*(2,86) = 2.417, *p* = ns. Also in contrast to other dependent measures, self-report of mind wandering was observed to be more frequent in high negative mood participants compared to low negative mood participants in this Experiment, *F*(1,43) = 6.754, *p* < 0.05. The interaction of stress and negative mood was not significant, *F*(1,43) = 1.664, *p* = ns, with no other effects observed, *F*s < 0.6.

Planned comparisons of high versus low negative mood participants in block 1 data were not significant, with mean differences opposite to those predicted for both high stress, *t*(20) = -1.247, *p* = ns, and low stress groups, *t*(23) = -2.616, *p* = ns.

#### Pupil dilation

Pupil diameter was observed to vary across block, with overall larger mean diameters in block 2 compared with block 1 and block 3, *F*(2,86) = 8.236, *p* < 0.01, with no significant linear trend, *F*(1,43) = 0.225, *p* = ns. Main effects of stress, *F*(1,43) = 2.682, *p* = ns, and negative mood, *F*(1,43) = 0.159, *p* = ns, were not significant. A significant interaction of stress and negative mood supported the observation of larger pupil diameters in high versus low negative mood participants in the high-stress condition, but the opposite of this pattern in the low stress condition, *F*(1,43) = 8.291, *p* < 0.01. This pattern of data appeared consistent over blocks, with no interaction of block by stress, *F*(2,86) = 1.678, *p* = ns, and no other effects, *F*s < 0.7.

Planned comparisons of block 1 data showed that in the high-stress group, high negative mood participants had larger pupil diameters compared to low negative mood participants, *t*(20) = 2.065, *p* < 0.05, one-tailed. This pattern was the opposite to predicted for the low stress group, with larger pupil diameters larger for low versus high negative affect participants, *t*(23) = -2.162, *p* < 0.5.

#### Retrospective report of thought content

The next series of analyses investigate whether the content of participants’ mind-wandering episodes differs between high and low negative mood participants in the high-stress condition, during the first experimental block. Descriptive statistics for the first experimental block are shown in **Table 1**. As per Experiment 1, retrospective thought content was analyzed using a Chi-square Test of Independence on the factors *Negative Mood* (low, high) as well as *Tense* (past, present, or future), *Valence* (positive, negative, or neutral), and *Focus* (task-related, TSST-related, and task-unrelated). The high mood group were more likely to entertain thoughts about the TSST relative to the low mood group, however, the trend just failed to reach significance, χ^2^(2) = 5.683, *p* = 0.058, *V* = 0.508. The effect of negative mood on retrospective report failed to reach significance for Tense, χ^2^(2) = 1.833, *p* = ns, *V* = 0.289, or Valence, χ^2^(2) = 2.640, *p* = ns, *V* = 0.346. Group differences failed to reach significance for the remaining factors (χ^2^ < 3).

### DISCUSSION

The goal of Experiment 2 was to replicate the trends observed in Experiment 1, with the inclusion of pupillometric evidence of resource availability, to develop a bio-cognitive account of stress on mind-wandering behavior. When exposed to our high-stress manipulation, participants in the high negative mood group displayed more variable response behavior and made more errors compared to participants with low negative mood, and showed relatively stable versus decreasing patterns of self-report mind wandering compared to other participants over time. These participants also exhibited larger pupil dilation on average relative to controls. These effects of mood were relatively absent in the low stress group, providing evidence for resource dependent, bio-cognitive account of stress on mind-wandering behavior.

## GENERAL DISCUSSION

The current work aims to investigate the effects of stress on mind-wandering behavior within both the cognitive-adaptive (Experiment 1) and bio-cognitive (Experiment 2) components of transactional theory. Participants were grouped based on their level of negative mood prior to experiment participation, and were randomly assigned to either the high stress or low stress version of the TSST. Participants were then required to complete the SART as a measure of mind-wandering propensity. With the current work representing the first investigation of stress effects on mind-wandering behavior, Experiment 1 was exploratory in nature with the minimal prediction that negative mood would potentiate the effects of stress. Predictions in Experiment 2 were bolstered by the trends observed in Experiment 1.

The cognitive-adaptive component of transactional theory predicts cognitive performance based on resource allocation (coping strategy) while the bio-cognitive component predicts performance based on resource availability. When both components are used in conjunction, transactional theory provides a comprehensive understanding of stress effects on cognition. Findings from Experiment 1 reveal that participants in the high negative mood group are more likely to disengage from the task (indicated by an increased response time variability and error rate) and think about the stressor when exposed to the high-stress condition relative to the low negative mood group. This stressor-oriented focus, reflecting an emotion-focused coping strategy, was observed during the first experimental block only and failed to replicate in the low stress condition, suggesting that the inattentive state observed was driven by the stress manipulation. This behavioral trend was observed in both experiments. Findings from Experiment 2 further reveal that participants in the high negative mood, high-stress condition also demonstrated reduced resource availability (larger pupil diameter) during the first block relative to the low negative mood group. Stress-induced limitations in resource availability promote the select processing of information that is most relevant to the self (Wells and Matthews, 1994; Chajut and Algom, 2003; Kofman et al., 2006). These data therefore suggest that acute stress limits resource availability in individuals with a negative disposition and, in the initiation of an emotion-focused coping strategy, promotes the select processing of stressor-oriented information at the expense of task performance.

Previous work on the relation between attentional state and pupil diameter support this interpretation, with pupillary construction associated with indices of task engagement and dilation with indices of task disengagement (Gilzenrat et al., 2010). Smallwood et al. (2011a) provide additional evidence for the systematic relationship between pupil diameter and attentional state using the SART, reporting pupillary dilation during mind-wandering episodes and constriction during task focus. Pupil dilation also reflects activation of the Locus Coeruleus-Norepinephrine (LC/NE) system (Yoshitomi et al., 1985; Koss, 1986; Loewenfeld, 1993; Frith and Frith, 2006). The LC/NE system is a prominent network within the hypothalamic–pituitary–adrenal axis (HPAA), a constellation of regions activated in stressful situations that operates to maintain homeostasis within the body (e.g., Woodward et al., 1991; Morilak et al., 2005). Acute stress has been shown to induce a phasic release of norepinephrine (NE) from the LC/NE system (e.g., Abercrombie and Jacobs, 1987a, b; Svensson, 1987; Cecchi et al., 2002a, b). In the attention literature, phasic release of NE enhances the processing of stimuli deemed to be of high reward and utility (Aston-Jones and Cohen, 2005). Previous work suggests that those in a depressive state are more likely to ruminate relative to healthy controls (for a review, see Salguero et al., 2012). Depressive rumination is intrusive and persistent (Lam et al., 2003; Wenzlaff and Luxton, 2003), and is often fixated on a social problem perceived by the individual to be severe (Edwards and Weary, 1993; Lyubomirsky et al., 1999; Treynor et al., 2003; Rudolph and Conley, 2005). In the face of a psychosocial stressor such as the TSST, it is possible that participants in a negative mood, who typically employ an emotion-focused coping strategy (Coyne et al., 1981; Parker and Brown, 1982), appraise stressor-focused thoughts to be of higher utility than the post-stressor SART and that phasic release of NE potentiates the internal focus of attention. As the HPAA feedback mechanisms terminate the stress response and phasic release of NE begins to diminish (Chamberlain et al., 2006), performance of participants with high levels of negative mood align with the low negative mood group and low stress controls. A stress-based mind-wandering paradigm therefore not only offers a unique framework for investigating the neurochemical basis of mind-wandering behavior, but may also provide a framework for investigating how depressive populations value information in situations of limited processing capacity.

Regardless of stress induction, depressive populations typically mind wander more than healthy controls (Smallwood et al., 2007, 2009; Smallwood and O’Connor, 2011). It would therefore be reasonable to predict that in the low stress condition, participants reporting a high level of negative mood would demonstrate a heightened propensity to mind wander relative to the low mood group. Instead, participants in the low negative mood group were more likely to mind wander (higher response time variability and errors rates) and have limited resource availability (larger pupil diameter) in the low stress condition, although this trend did fail to reach significance for all dependent measures. This trend validates the current interpretation that mood interacts with stress to promote mind-wandering behavior, but raises the question of why participants with low negative mood would mind wander more than high negative mood participants in circumstances of low stress. One possibility could be that the low stress condition induces a positive state in those with a depressive mood. Fredrickson and Levenson (1998) and Fredrickson et al. (1999) argue that positive mood can regulate negative mood, with positive mood having been linked to a broadened scope of attention and heightened resource availability (for a review, see Fredrickson, 1998). Eliciting positive emotions has even been shown to undo the effects of negative mood (Cabanac, 1971; Solomon, 1980; Nezu et al., 1988). In the low stress condition used in the current work, participants are asked to spend 5 min talking about a favorite experience or movie. While speculative, it is possible that the low stress condition temporarily alleviates depressive mood and promotes an on-task focus in high negative mood participants. Stress-related research typically utilizes the high-stress version of the TSST, and transactional models typically focus on the interaction between high-stress situations and mood. Findings in the current work therefore offer a new route of investigation within the stress literature: does the low stress version of the TSST (engaging in positive thought) induce a focused state of attention in depressive populations?

While the low stress condition may elicit positive moods in participants with high negative mood, Schoofs et al. (2008) found that exposure to the high-stress version of the TSST can induce a negative mood in participants. The current work fails to include measures of TSST effectiveness beyond behavioral measures, and therefore it could be argued that the observed effects are an artifact of temporary mood induction. Indeed, the behavioral deficits observed during the first block are congruent with previous reports of attentional deficits following negative mood induction (Smallwood and O’Connor, 2011). The inclusion of a post-TSST mood questionnaire or retrospective report of stressor effectiveness would alleviate the current limitation. However, in the interest to investigate the depleting effect of stress on task inattention, increasing the time interval between the TSST and task performance was perceived as a threat to the validity of data interpretation. In addition to the passage of time, it is possible that having participants actively reflect on their current mood may interfere with the stress manipulation. Investigating the mediating role of stress-induced mood on cognition, rather than the mediating role of mood on stress effects (as in the current work), would be an interesting route of investigation and may help shed light on the mechanisms underlying the effect of stress on mind wandering.

Introducing a time interval between the TSST and SART performance may also influence the degree to which participants are likely to report of mind wandering during the first experimental block. In the current work, the high and low negative mood groups were equally likely to report off-task thought in the high-stress condition, even though behavioral measures of task inattention appear to differ between groups. One reason for this finding could be stereotype threat induced by the TSST. Stereotype threat refers to the psychosocial phenomena whereby an individual feels under threat of a stigmatized social identity (Steele and Aronson, 1995; Steele, 1997), with the individual maintaining an effort to sustain a self-image of competence beyond race, gender, socioeconomic status, or age (Steele, 1988; Aronson et al., 1999). The TSST is designed to stimulate a manageable level of stress through social evaluative threat and perceived uncontrollability (Kirschbaum et al., 1993), providing the ideal environmental context for facilitation of stereotype threat. In light of evidence that stereotype threat facilitates worries related to perceived task performance (Cadinu et al., 2005; Beilock et al., 2007), it is reasonable to presume that participants were likely to diminish the degree to which they reported mind-wandering behavior in the high-stress condition, regardless of negative mood. This interpretation is also congruent with previous reports that self-report measures are vulnerable to threats of performance evaluation (Vinski and Watter, 2012), and the failure to observe consistent correlations between self-report and the remaining dependent measures across both experiments. Future research on the effects of stress using the mind-wandering paradigm might benefit from measuring the degree to which participants felt intimidated by the high-stress condition of the TSST as a mediator of self-report measures.

The current findings are interpreted with a focus on the detrimental effects of stress on participants that report a high level of negative mood. However, an alternative interpretation could be that low levels of negative mood buffers against the detrimental effects of stress, resulting in enhanced task-focus immediately following stress induction. This interpretation is congruent with previous stress-related work in non-depressive populations that demonstrates improved vigilance at moderate levels of arousal (for a review, see Warm et al., 2008). This interpretation would also account for the steady decline in vigilance (and increase in mind wandering frequency) as the effects of stress diminish over time. However, participants with low levels of negative mood were equally likely to engage in mind-wandering behavior regardless of the stress condition across all experimental blocks, and mind wandering typically increases throughout task duration regardless of participants’ mood due to a decline in available cognitive resources necessary to maintain stimulus-independent thought (Teasdale et al., 1995). The current experiments do not permit the ability to tease apart whether the temporary low levels of mind wandering observed in the low negative mood, high-stress condition is due to the effects of stress or due to a natural task-oriented focus of attention at the early stages of experiment participation typically observed in non-depressive populations, however, this route of investigation would be interesting in future studies of stress effects on mind wandering.

While we discuss the current effects within a theoretical framework of attentional control, it is possible that other causal arrangements could account for the observed effects. For example, the difference in self-report of on-task performance over blocks could more causally relate to the differences we observe in behavioral performance measures. Our view is that all of these measures reflect aspects of attentional control, but concede that it is not possible from our data to properly tease apart potentially separate effects of block and more basic attentional effects.

While *a priori* predictions were replicated between the two experiments, it is imperative to consider the findings that failed to replicate, including the main effects of stress and negative mood. One possibility for the discrepant findings could be the difference in sample size: Experiment 1 has a sample size almost twice the size of Experiment 2. It is therefore entirely plausible that the power of the observed effects in Experiment 1 is not large enough to translate into a smaller sample. It is also plausible that including an eye-tracker in Experiment 2 introduced a degree of discomfort for participants, which may have diminished the difference in mind-wandering behavior originally observed between groups in Experiment 1.

In summary, findings in the current work provide evidence for a transactional account of stress on mind-wandering behavior. Specifically, acute stress appears to limit the availability of cognitive resources in participants that report a high level of negative mood (Experiment 2) and, in the alongside the initiation of an emotion-focused coping strategy, is likely to induce a temporary stressor-oriented focus of attention at the expense of task performance (Experiment 1).

## Conflict of Interest Statement

The authors declare that the research was conducted in the absence of any commercial or financial relationships that could be construed as a potential conflict of interest.
